# *N*-(1,4-Dioxo-1,4-dihydro­naphthalen-2-yl)benzamide

**DOI:** 10.1107/S1600536812034150

**Published:** 2012-08-25

**Authors:** Yakini Brandy, Ray J. Butcher, Oladapo Bakare

**Affiliations:** a Department of Chemistry, Howard University, 525 College Street NW, Washington, DC 20059, USA

## Abstract

The title compound, C_17_H_11_NO_3_, was an inter­mediate synthesized during bis­acyl­ation of 2-amino-1,4-naphtho­quinone with benzoyl chloride. A mixture of block- and needle-shaped crystals were obtained after column chromatography. The block-shaped crystals were identified as the imide and the needles were the title amide. The naphtho­quinone scaffold is roughly planar (r.m.s. deviation = 0.047 Å for the C atoms). The N—H and C=O bonds of the amide group are *anti* to each other. A dihedral angle between the naphtho­quinone ring system and the amide group of 3.56 (3)°, accompanied by a dihedral angle between the amide group and the phenyl group of 9.51 (3)°, makes the naphtho­quinone ring essentially coplanar with the phenyl ring [dihedral angle = 7.12 (1)°]. In the crystal, molecules are linked by a weak N—H⋯O hydrogen bond and by two weak C—H⋯O interactions leading to the formation of zigzag chains along [010].

## Related literature

For similar crystal structures, see: Brandy *et al.* (2009 ▶, 2012 ▶); Akinboye *et al.* (2009*a* ▶,*b* ▶). For the pharmacological properties of related compounds, see: Bakare *et al.* (2003 ▶); Berhe *et al.* (2008 ▶); Lien *et al.* (1997 ▶); Huang, *et al.* (2005 ▶); Khraiwesh *et al.* (2011 ▶).
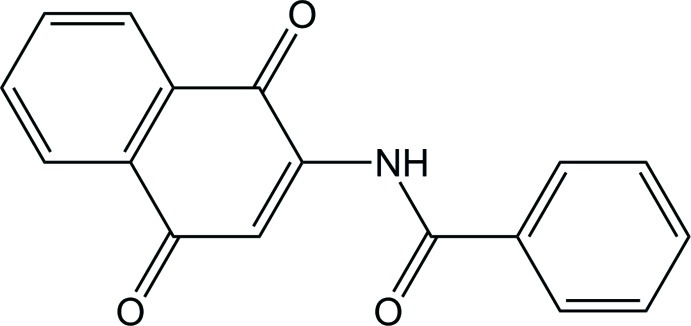


## Experimental

### 

#### Crystal data

C_17_H_11_NO_3_
*M* *_r_* = 277.27
Monoclinic, 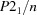

*a* = 6.9433 (3) Å
*b* = 12.0112 (4) Å
*c* = 15.2129 (5) Å
β = 94.129 (3)°
*V* = 1265.42 (8) Å^3^
*Z* = 4
Cu *K*α radiation
μ = 0.83 mm^−1^
*T* = 123 K
0.67 × 0.12 × 0.08 mm

#### Data collection

Oxford Diffraction Xcalibur Ruby Gemini diffractometer
Absorption correction: multi-scan (*CrysAlis PRO*; Oxford Diffraction, 2007 ▶) *T*_min_ = 0.819, *T*_max_ = 1.000
4550 measured reflections
2553 independent reflections
2123 reflections with *I* > 2σ(*I*)
*R*_int_ = 0.037

#### Refinement

*R*[*F* ^2^ > 2σ(*F* ^2^)] = 0.052
*wR*(*F* ^2^) = 0.148
*S* = 1.05
2553 reflections
190 parameters
H-atom parameters constrained
Δρ_max_ = 0.25 e Å^−3^
Δρ_min_ = −0.25 e Å^−3^

### 

Data collection: *CrysAlis PRO* (Oxford Diffraction, 2007 ▶); cell refinement: *CrysAlis PRO*; data reduction: *CrysAlis PRO*; program(s) used to solve structure: *SHELXS97* (Sheldrick, 2008 ▶); program(s) used to refine structure: *SHELXL97* (Sheldrick, 2008 ▶); molecular graphics: *SHELXTL* (Sheldrick, 2008 ▶); software used to prepare material for publication: *SHELXTL*.

## Supplementary Material

Crystal structure: contains datablock(s) I, global. DOI: 10.1107/S1600536812034150/bt5965sup1.cif

Structure factors: contains datablock(s) I. DOI: 10.1107/S1600536812034150/bt5965Isup2.hkl

Supplementary material file. DOI: 10.1107/S1600536812034150/bt5965Isup3.cml

Additional supplementary materials:  crystallographic information; 3D view; checkCIF report

## Figures and Tables

**Table 1:** Hydrogen-bond geometry (Å, °)

*D*—H⋯*A*	*D*—H	H⋯*A*	*D*⋯*A*	*D*—H⋯*A*
N1—H1*A*⋯O2^i^	0.88	2.65	3.3299 (19)	135
C4—H4*A*⋯O3^i^	0.95	2.49	3.149 (2)	127
C17—H17*A*⋯O2^i^	0.95	2.57	3.404 (2)	146

Symmetry code: (i) 
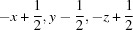
.

**Fig. 1.**
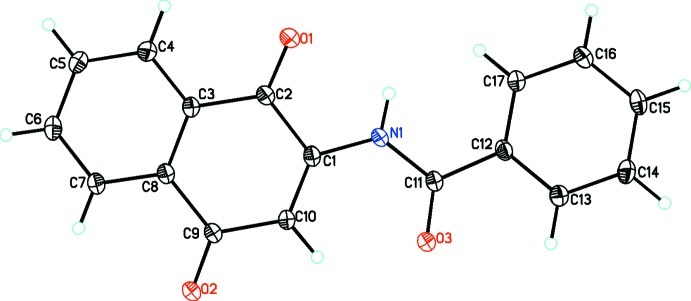
Diagram of C17H11NO3 showing the atom labeling. Displacement ellipsoids are at the 30% probability level.

**Fig. 2.**
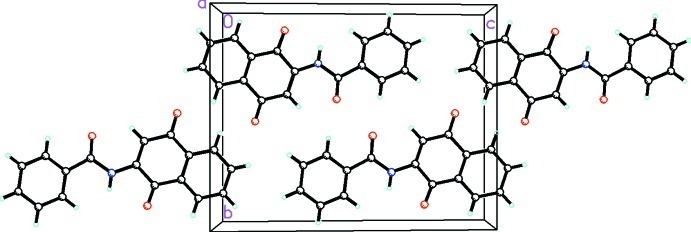
The molecular packing for C17H11NO3 viewed along the a axis.

## supplementary crystallographic information

### Comment

Amido and imidonaphthoquinones are known for their anti-inflammatory, antiplatelet, antiallergic, antiparasitic and anticancer activities (Lien *et al.*(1997); Huang *et al.* (2005); Bakare *et al.* (2003), Khraiwesh *et al.* (2011)). During imide synthesis (2-*N*-bis(benzoyl)amino-1,4-naphthoquinone (Brandy *et al.* (2012)), we obtained the intermediate amido analog, 2-*N*-benzoylamino-1,4-naphthoquinone. In order to eventually perform an anticancer SAR (structure-activity relationship) study, these intermediate amido analogs were isolated, crystallized and subjected to an X-ray diffraction study.

This showed that the naphthoquinone scaffold was planar with N—H and C=O bonds anti to each other. A dihedral angle between the naphthoquinone ring and the amide group of -2.6 (3)°, accompanied with the dihedral angle between the amide group and the phenyl group of -8.8 (2)° makes the naphthoquinone ring coplanar to the phenyl group. The bond distances and angles are similar to those found in related structures (Brandy *et al.*, 2009, 2012; Akinboye *et al.*, 2009*a*, 2009*b*). The crystal packing pattern results from N—H···O hydrogen bonds along with two weak intermolecular C—H···O interactions.

### Experimental

2-Amino-1,4-naphthoquinone (318 mg, 1.83 mmol) was dissolved in freshly distilled THF (15 ml). NaH (115 mg, 4.78 mmol) was added and the mixture was stirred at room temperature for 15 min. The appropriate benzoyl chloride (0.55 ml, 4.74 mmol) was added, drop wise, and the mixture was stirred for 24 h. THF was evaporated under vacuum and the mixture was washed with ice-water (10 g ice in 10 ml water). The ice-water mixture was extracted with CH_2_Cl_2_ (30 ml, 20 ml consecutively) and the combined organic phase washed with water (3 *x* 20 ml), saturated NaCl solution (20 ml), then dried over anhydrous MgSO_4_. The crude was purified *via* triturating in ethanol (2 ml) and column chromatography with an eluent mixture of ethyl acetate and hexane to furnish the amide (39 mg, 7.7%). A mixure of block and needle crystals were obtained from column chromatography. The block crystals were identified as the imide (Brandy *et al.*, 2012) and the needles were identified as the amide. The needle crystals were hand-picked from the mixture and analyzed by X-ray diffraction.

### Refinement

H atoms were placed in geometrically idealized positions and constrained to ride on their parent atoms with N—H = 0.88Å and C—H = 0.95Å and *U*_iso_(H) = 1.2*U*_eq_(C,N).

### Crystal data

**Table:**

C_17_H_11_NO_3_	*F*(000) = 576
*M**_r_* = 277.27	*D*_x_ = 1.455 Mg m^−^^3^
Monoclinic, *P*2_1_/*n*	Cu *K*α radiation, λ = 1.54178 Å
*a* = 6.9433 (3) Å	Cell parameters from 1585 reflections
*b* = 12.0112 (4) Å	θ = 2.9–75.6°
*c* = 15.2129 (5) Å	µ = 0.83 mm^−^^1^
β = 94.129 (3)°	*T* = 123 K
*V* = 1265.42 (8) Å^3^	Needle, pale yellow orange
*Z* = 4	0.67 × 0.12 × 0.08 mm

### Data collection

**Table:**

Oxford Diffraction Xcalibur Ruby Gemini diffractometer	2553 independent reflections
Radiation source: Enhance (Cu) X-ray Source	2123 reflections with *I* > 2σ(*I*)
Graphite monochromator	*R*_int_ = 0.037
Detector resolution: 10.5081 pixels mm^-1^	θ_max_ = 75.7°, θ_min_ = 4.7°
ω scans	*h* = −7→8
Absorption correction: multi-scan (*CrysAlis PRO*; Oxford Diffraction, 2007)	*k* = −14→14
*T*_min_ = 0.819, *T*_max_ = 1.000	*l* = −18→18
4550 measured reflections	

### Refinement

**Table:**

Refinement on *F*^2^	Primary atom site location: structure-invariant direct methods
Least-squares matrix: full	Secondary atom site location: difference Fourier map
*R*[*F*^2^ > 2σ(*F*^2^)] = 0.052	Hydrogen site location: inferred from neighbouring sites
*wR*(*F*^2^) = 0.148	H-atom parameters constrained
*S* = 1.05	*w* = 1/[σ^2^(*F*_o_^2^) + (0.0937*P*)^2^] where *P* = (*F*_o_^2^ + 2*F*_c_^2^)/3
2553 reflections	(Δ/σ)_max_ < 0.001
190 parameters	Δρ_max_ = 0.25 e Å^−^^3^
0 restraints	Δρ_min_ = −0.25 e Å^−^^3^

### Special details

**Table:**

Geometry. All e.s.d.'s (except the e.s.d. in the dihedral angle between two l.s. planes) are estimated using the full covariance matrix. The cell e.s.d.'s are taken into account individually in the estimation of e.s.d.'s in distances, angles and torsion angles; correlations between e.s.d.'s in cell parameters are only used when they are defined by crystal symmetry. An approximate (isotropic) treatment of cell e.s.d.'s is used for estimating e.s.d.'s involving l.s. planes.
Refinement. Refinement of *F*^2^ against ALL reflections. The weighted *R*-factor *wR* and goodness of fit *S* are based on *F*^2^, conventional *R*-factors *R* are based on *F*, with *F* set to zero for negative *F*^2^. The threshold expression of *F*^2^ > σ(*F*^2^) is used only for calculating *R*-factors(gt) *etc*. and is not relevant to the choice of reflections for refinement. *R*-factors based on *F*^2^ are statistically about twice as large as those based on *F*, and *R*- factors based on ALL data will be even larger.

### Fractional atomic coordinates and isotropic or equivalent isotropic displacement parameters (Å^2^)

**Table:**

	*x*	*y*	*z*	*U*_iso_*/*U*_eq_	
O1	0.4411 (2)	0.09807 (11)	0.24463 (8)	0.0373 (3)	
O2	0.3847 (2)	0.51857 (10)	0.13486 (8)	0.0348 (3)	
O3	0.3674 (2)	0.41588 (11)	0.43416 (8)	0.0354 (3)	
N1	0.3825 (2)	0.24883 (12)	0.36581 (9)	0.0273 (3)	
H1A	0.3821	0.1763	0.3741	0.033*	
C1	0.3905 (2)	0.28621 (14)	0.27989 (10)	0.0253 (4)	
C2	0.4119 (2)	0.19196 (14)	0.21661 (10)	0.0269 (4)	
C3	0.3957 (2)	0.21822 (14)	0.12102 (10)	0.0252 (4)	
C4	0.3886 (2)	0.13177 (15)	0.05992 (11)	0.0299 (4)	
H4A	0.3960	0.0566	0.0793	0.036*	
C5	0.3707 (2)	0.15588 (15)	−0.02938 (11)	0.0314 (4)	
H5A	0.3629	0.0971	−0.0713	0.038*	
C6	0.3641 (2)	0.26586 (16)	−0.05777 (11)	0.0320 (4)	
H6A	0.3547	0.2820	−0.1191	0.038*	
C7	0.3713 (2)	0.35219 (15)	0.00297 (11)	0.0290 (4)	
H7A	0.3670	0.4272	−0.0168	0.035*	
C8	0.3848 (2)	0.32888 (14)	0.09291 (10)	0.0251 (4)	
C9	0.3872 (2)	0.42102 (14)	0.15843 (10)	0.0266 (4)	
C10	0.3855 (2)	0.39248 (14)	0.25204 (10)	0.0270 (4)	
H10A	0.3807	0.4506	0.2942	0.032*	
C11	0.3750 (2)	0.31531 (14)	0.43956 (10)	0.0261 (3)	
C12	0.3814 (2)	0.25771 (14)	0.52731 (10)	0.0252 (4)	
C13	0.4058 (2)	0.32638 (15)	0.60106 (11)	0.0294 (4)	
H13A	0.4142	0.4047	0.5938	0.035*	
C14	0.4181 (3)	0.28079 (16)	0.68528 (11)	0.0324 (4)	
H14A	0.4353	0.3279	0.7354	0.039*	
C15	0.4050 (2)	0.16642 (16)	0.69612 (11)	0.0316 (4)	
H15A	0.4137	0.1352	0.7536	0.038*	
C16	0.3794 (2)	0.09777 (15)	0.62296 (11)	0.0308 (4)	
H16A	0.3694	0.0195	0.6306	0.037*	
C17	0.3681 (2)	0.14279 (15)	0.53839 (11)	0.0286 (4)	
H17A	0.3514	0.0954	0.4884	0.034*	

### Atomic displacement parameters (Å^2^)

**Table:**

	*U*^11^	*U*^22^	*U*^33^	*U*^12^	*U*^13^	*U*^23^
O1	0.0618 (8)	0.0246 (7)	0.0265 (6)	0.0064 (6)	0.0101 (5)	0.0045 (5)
O2	0.0576 (7)	0.0236 (6)	0.0238 (6)	−0.0019 (5)	0.0080 (5)	0.0045 (5)
O3	0.0604 (8)	0.0235 (6)	0.0221 (6)	0.0007 (5)	0.0030 (5)	0.0025 (5)
N1	0.0412 (7)	0.0216 (7)	0.0196 (7)	0.0017 (5)	0.0047 (5)	0.0045 (5)
C1	0.0299 (7)	0.0271 (8)	0.0192 (7)	0.0005 (6)	0.0037 (5)	0.0022 (6)
C2	0.0352 (7)	0.0234 (8)	0.0226 (8)	0.0011 (6)	0.0057 (6)	0.0038 (6)
C3	0.0302 (7)	0.0258 (8)	0.0202 (7)	−0.0002 (6)	0.0059 (5)	0.0011 (6)
C4	0.0380 (8)	0.0253 (8)	0.0271 (8)	−0.0008 (6)	0.0084 (6)	−0.0007 (6)
C5	0.0392 (8)	0.0315 (9)	0.0241 (8)	0.0001 (7)	0.0060 (6)	−0.0057 (7)
C6	0.0365 (8)	0.0393 (10)	0.0208 (7)	0.0005 (7)	0.0057 (6)	0.0008 (7)
C7	0.0369 (8)	0.0294 (9)	0.0211 (8)	0.0007 (7)	0.0054 (6)	0.0035 (6)
C8	0.0288 (7)	0.0260 (9)	0.0208 (7)	0.0001 (6)	0.0042 (5)	0.0025 (6)
C9	0.0349 (7)	0.0239 (8)	0.0214 (7)	−0.0006 (6)	0.0043 (6)	0.0037 (6)
C10	0.0378 (8)	0.0250 (8)	0.0184 (7)	−0.0014 (6)	0.0041 (6)	0.0010 (6)
C11	0.0325 (7)	0.0265 (8)	0.0195 (7)	0.0004 (6)	0.0027 (6)	0.0029 (6)
C12	0.0289 (7)	0.0270 (8)	0.0201 (8)	0.0007 (6)	0.0037 (6)	0.0046 (6)
C13	0.0373 (8)	0.0286 (9)	0.0227 (8)	0.0001 (6)	0.0045 (6)	0.0017 (6)
C14	0.0403 (9)	0.0382 (10)	0.0191 (7)	−0.0006 (7)	0.0048 (6)	−0.0015 (6)
C15	0.0351 (8)	0.0412 (10)	0.0187 (7)	0.0001 (7)	0.0032 (6)	0.0077 (7)
C16	0.0390 (8)	0.0287 (9)	0.0247 (8)	−0.0003 (7)	0.0034 (6)	0.0070 (7)
C17	0.0373 (8)	0.0282 (9)	0.0202 (7)	0.0002 (6)	0.0024 (6)	0.0017 (6)

### Geometric parameters (Å, º)

**Table:**

O1—C2	1.217 (2)	C7—C8	1.393 (2)
O2—C9	1.225 (2)	C7—H7A	0.9500
O3—C11	1.212 (2)	C8—C9	1.489 (2)
N1—C11	1.381 (2)	C9—C10	1.466 (2)
N1—C1	1.3869 (19)	C10—H10A	0.9500
N1—H1A	0.8800	C11—C12	1.501 (2)
C1—C10	1.345 (2)	C12—C13	1.393 (2)
C1—C2	1.500 (2)	C12—C17	1.394 (2)
C2—C3	1.484 (2)	C13—C14	1.390 (2)
C3—C4	1.392 (2)	C13—H13A	0.9500
C3—C8	1.397 (2)	C14—C15	1.387 (3)
C4—C5	1.386 (2)	C14—H14A	0.9500
C4—H4A	0.9500	C15—C16	1.386 (3)
C5—C6	1.390 (3)	C15—H15A	0.9500
C5—H5A	0.9500	C16—C17	1.393 (2)
C6—C7	1.387 (2)	C16—H16A	0.9500
C6—H6A	0.9500	C17—H17A	0.9500
			
C11—N1—C1	125.79 (14)	O2—C9—C10	120.48 (15)
C11—N1—H1A	117.1	O2—C9—C8	121.05 (14)
C1—N1—H1A	117.1	C10—C9—C8	118.44 (14)
C10—C1—N1	127.04 (15)	C1—C10—C9	121.76 (15)
C10—C1—C2	121.04 (14)	C1—C10—H10A	119.1
N1—C1—C2	111.90 (14)	C9—C10—H10A	119.1
O1—C2—C3	122.60 (16)	O3—C11—N1	121.69 (14)
O1—C2—C1	119.73 (14)	O3—C11—C12	121.20 (15)
C3—C2—C1	117.67 (14)	N1—C11—C12	117.10 (15)
C4—C3—C8	120.45 (15)	C13—C12—C17	119.61 (14)
C4—C3—C2	119.49 (15)	C13—C12—C11	115.94 (15)
C8—C3—C2	120.05 (15)	C17—C12—C11	124.45 (15)
C5—C4—C3	119.67 (16)	C14—C13—C12	120.32 (16)
C5—C4—H4A	120.2	C14—C13—H13A	119.8
C3—C4—H4A	120.2	C12—C13—H13A	119.8
C4—C5—C6	120.13 (16)	C15—C14—C13	119.96 (16)
C4—C5—H5A	119.9	C15—C14—H14A	120.0
C6—C5—H5A	119.9	C13—C14—H14A	120.0
C7—C6—C5	120.31 (15)	C16—C15—C14	119.95 (15)
C7—C6—H6A	119.8	C16—C15—H15A	120.0
C5—C6—H6A	119.8	C14—C15—H15A	120.0
C6—C7—C8	120.04 (16)	C15—C16—C17	120.41 (17)
C6—C7—H7A	120.0	C15—C16—H16A	119.8
C8—C7—H7A	120.0	C17—C16—H16A	119.8
C7—C8—C3	119.36 (16)	C16—C17—C12	119.75 (16)
C7—C8—C9	120.32 (15)	C16—C17—H17A	120.1
C3—C8—C9	120.32 (14)	C12—C17—H17A	120.1
			
C11—N1—C1—C10	−2.6 (3)	C3—C8—C9—O2	177.33 (15)
C11—N1—C1—C2	175.82 (14)	C7—C8—C9—C10	175.06 (14)
C10—C1—C2—O1	170.53 (16)	C3—C8—C9—C10	−4.8 (2)
N1—C1—C2—O1	−8.0 (2)	N1—C1—C10—C9	−177.44 (15)
C10—C1—C2—C3	−9.6 (2)	C2—C1—C10—C9	4.3 (2)
N1—C1—C2—C3	171.88 (13)	O2—C9—C10—C1	−179.15 (15)
O1—C2—C3—C4	8.5 (2)	C8—C9—C10—C1	3.0 (2)
C1—C2—C3—C4	−171.42 (14)	C1—N1—C11—O3	2.9 (3)
O1—C2—C3—C8	−172.50 (15)	C1—N1—C11—C12	−175.70 (14)
C1—C2—C3—C8	7.6 (2)	O3—C11—C12—C13	−8.8 (2)
C8—C3—C4—C5	0.1 (2)	N1—C11—C12—C13	169.78 (14)
C2—C3—C4—C5	179.07 (15)	O3—C11—C12—C17	172.36 (16)
C3—C4—C5—C6	1.4 (3)	N1—C11—C12—C17	−9.0 (2)
C4—C5—C6—C7	−1.4 (2)	C17—C12—C13—C14	0.4 (2)
C5—C6—C7—C8	−0.1 (2)	C11—C12—C13—C14	−178.52 (15)
C6—C7—C8—C3	1.6 (2)	C12—C13—C14—C15	−0.3 (2)
C6—C7—C8—C9	−178.34 (14)	C13—C14—C15—C16	−0.2 (3)
C4—C3—C8—C7	−1.5 (2)	C14—C15—C16—C17	0.5 (3)
C2—C3—C8—C7	179.46 (14)	C15—C16—C17—C12	−0.5 (2)
C4—C3—C8—C9	178.37 (14)	C13—C12—C17—C16	0.0 (2)
C2—C3—C8—C9	−0.6 (2)	C11—C12—C17—C16	178.79 (15)
C7—C8—C9—O2	−2.8 (2)		

### Hydrogen-bond geometry (Å, º)

**Table:**

*D*—H···*A*	*D*—H	H···*A*	*D*···*A*	*D*—H···*A*
N1—H1*A*···O2^i^	0.88	2.65	3.3299 (19)	135
C4—H4*A*···O3^i^	0.95	2.49	3.149 (2)	127
C17—H17*A*···O2^i^	0.95	2.57	3.404 (2)	146

Symmetry code: (i) −*x*+1/2, *y*−1/2, −*z*+1/2.
